# Genetic Basis for Variation of Metalloproteinase-Associated Biochemical Activity in Venom of the Mojave Rattlesnake (*Crotalus scutulatus scutulatus*)

**DOI:** 10.1155/2013/251474

**Published:** 2013-07-29

**Authors:** Ruben K. Dagda, Sardar Gasanov, Ysidro De La OIII, Eppie D. Rael, Carl S. Lieb

**Affiliations:** ^1^Pharmacology Department, University of Nevada School of Medicine, Manville Building 19A, Reno, NV 89557, USA; ^2^Science Department, Tashkent Ulugbek International School, 5-A J. Shoshiy Street, 100100 Tashkent, Uzbekistan; ^3^Department of Biological Sciences, University of Texas at El Paso, 500 West University Avenue, El Paso, TX 79968, USA

## Abstract

The metalloproteinase composition and biochemical profiles of rattlesnake venom can be highly variable among rattlesnakes of the same species. We have previously shown that the neurotoxic properties of the Mojave rattlesnake (*Crotalus scutulatus scutulatus*) are associated with the presence of the Mojave toxin A subunit suggesting the existence of a genetic basis for rattlesnake venom composition. In this report, we hypothesized the existence of a genetic basis for intraspecies variation in metalloproteinase-associated biochemical properties of rattlesnake venom of the Mojave rattlesnake. To address this question, we PCR-amplified and compared the genomic DNA nucleotide sequences that code for the mature metalloproteinase domain of fourteen Mojave rattlesnakes captured from different geographical locations across the southwest region of the United States. In addition, the venoms from the same rattlesnakes were tested for their ability to hydrolyze fibrinogen, fibrin, casein, and hide powder azure and for induction of hemorrhage in mice. Overall, based on genomic sequencing and biochemical data, we classified Mojave rattlesnake venom into four distinct groups of metalloproteinases. These findings indicate that differences in nucleotide sequences encoding the mature proteinase domain and noncoding regions contribute to differences in venom metalloproteinase activities among rattlesnakes of the same species.

## 1. Introduction

Rattlesnake venom metalloproteinases are zinc-dependent enzymes that hydrolyze fibrin and fibrinogen, inactivate complement proteins, and promote hemorrhage *in vivo* [1–4]. In addition, rattlesnake venom metalloproteinases show differences in substrate specificity, proteolytic activity, molecular weight, and composition of structural domains [5, 6].

Rattlesnake venom metalloproteinases are members of the Reprolysin superfamily of metalloproteinases [7]. These venom enzymes have been subcategorized into four classes (P-I to P-IV) based on the differences in structural domains, molecular weight, and biochemical properties [7, 8]. The P-I group of rattlesnake venom metalloproteinases contains a proteinase domain, whereas the P-II group contains an additional disintegrin-like domain. The P-III group has an additional disintegrin-like domain and cysteine-rich sequence, whereas the P-IV group has an additional lectin-like sequence [8].

The P-II and P-III classes of rattlesnake venom metalloproteinases are one- to twofold more potent at inducing hemorrhage than the P-I metalloproteinases [8–11]. This observation suggests that the disintegrin-like and cysteine-rich domains play a critical role in inducing hemorrhage. In addition, the P-I metalloproteinases can be further divided into two subclasses, P-IA and P-IB. The P-IA subclass exhibits strong hemorrhagic activity, whereas the P-IB subclass has little or no hemorrhagic activity [12]. This observation implies that the P-IA metalloproteinases have certain structural features that are distinct from the disintegrin and cysteine-rich domains for inducing hemorrhage [13].

Rattlesnake venoms differ significantly in metalloproteinase composition [6, 14–16]. We have shown previously that rattlesnakes of the same species show differences in metalloproteinase composition and metalloproteinase-associated biochemical activities [4, 6, 14]. Various posttranslational modifications of metalloproteinases may contribute to differences in biochemical properties of rattlesnake venom [17]. For instance, the venom of the Mojave rattlesnake shows differences in hemorrhage induction and proteolytic activities, which are associated with the geographic location where the rattlesnake was captured. Indeed, Mojave rattlesnakes from Texas either lack or contain modest hemorrhagic activity, while Mojave rattlesnakes from central Arizona contain high hemorrhagic activity and to the same extent as venom from Western Diamondback (*C. atrox*) and Northern Blacktailed (*C. m. molossus*) rattlesnakes [16]. In further support of this observation, we have previously reported significant intraspecies differences in the neurotoxic properties of rattlesnake venom of the Mojave rattlesnake (*C. s. scutulatus*) and Southern Pacific rattlesnake (*C. helleri*). These data suggest that both genetics and environmental factors contribute to intraspecies variation in rattlesnake venom composition and their biochemical properties [18, 19]. A recent proteomics study of rattlesnake venom derived from twenty-one Mojave rattlesnakes showed an inverse relationship in the content of the Mojave toxin compared to snake venom metalloproteinase content which also correlated with geographical distribution of rattlesnakes [20]. 

In this study, we hypothesized that a genetic basis for intra-species biochemical variation of metalloproteinase activity exists in *C. s. scutulatus*. To address this question, we sequenced the proteinase domains of the metalloproteinase genes in fourteen Mojave rattlesnakes captured from different geographic locations of the southwest region of the United States. We did a battery of biochemical tests that evaluated metalloproteinase-associated proteolytic activity, nonmetalloproteinase proteolytic activity (esterases and non-zinc dependent serine proteases), and hemorrhagic activity. In summary, we categorized rattlesnake venom into four different groups based on their genomic DNA sequence homology and biochemical properties. Interestingly, the metalloproteinase gene of rattlesnake venom that lacked hemorrhagic activity contains a larger intronic region located within the proteinase domain and showed lower homology for the coding regions of the proteinase domain compared to the other groups of rattlesnake venom metalloproteinases. These observations suggest that differences in the noncoding region and single nucleotide polymorphisms located in the mature proteinase domain contribute to intra-species diversity in rattlesnake venom metalloproteinase composition.

## 2. Materials and Methods

### 2.1. Study Sample

 Mojave rattlesnake venoms were obtained from snakes captured in Arizona, New Mexico, and Texas in the United States. The geographical information of the rattlesnakes is shown in Table 1.

### 2.2. DNA Extraction

Blood was withdrawn from the caudal vein of the rattlesnake and transferred to a tube containing sodium citrate to prevent coagulation. DNA was extracted from whole blood using the DNA Zol reagent, a guanidine detergent (Molecular Research Center, Inc., Cincinnati, OH, USA). After precipitation with isopropanol and washing with ethanol, the DNA was stored in 0.01 M Tris-EDTA (TE) buffer. Quantification of DNA was done at OD260 nm.

### 2.3. Oligonucleotides

Primers for amplifying the metalloproteinase genes were designed according to published cDNA sequences for snake venom metalloproteinases [7, 21, 22]. Primers were designed to amplify the mature proteinase domain by annealing to conserved regions within the zymogen and the spacer/disintegrin regions as verified through BLAST (National Center for Biotechnology). The primer pair, amplification sites, and corresponding rattlesnake venom metalloproteinase sequences are shown in Table 2.

### 2.4. PCR

DNA amplification was done using buffer C PCR kit (Invitrogen, Carlsbad, CA, USA). DNA (1-2 *μ*g), primers (1 *μ*M), *Taq* DNA polymerase (2.5 units), dNTP mix (1 mM), and MgCl_2_ (2.5 mM) were mixed in a total volume of 50 *μ*L. PCR was done using the touchdown PCR method. In brief, the first seven cycles of PCR were done at a heat denaturation step of 95°C for 25 sec, an annealing step at 55°C for 30 sec, and an extension step at 72°C for 1 min, which were followed by 32 cycles of PCR at a heat denaturation step at 95°C for 25 sec, an annealing step at 55°C for 30 sec, and an extension step at 45°C for 1 min. The analysis of the amplified products was done by subjecting DNA products to electrophoresis in 1% agarose gels soaked in TAE (Tris-acetic acid-EDTA) buffer and visualizing with ethidium bromide when exposed in a UV box. A 100 base pair ladder (Amersham Pharmacia Biotech Inc.) was used to determine the size of products.

### 2.5. Cloning

PCR products were purified from the gel using the QIAquick Gel Extraction Kit (QIAGEN Inc., Valencia, CA, USA) and ligated into the pCR2.1 plasmid using the Original TA Cloning kit (Invitrogen, Carlsbad, CA, USA). The *E*. *coli* strain TOP10F′ competent cells were then transformed with DNA ligation reactions. Cells were grown overnight at 37°C on TSB plates containing kanamycin (100 *μ*g/mL), IPTG (0.1 M), and X-Gal (25 *μ*g/1.25 mL). White colonies were replated and grown overnight to ensure that the colonies were transformants. Positive clones were grown overnight at 37°C in 10 mL of TSB broth. Plasmids were isolated from the culture using the Wizard Plus Minipreps DNA Purification kit (Promega, madison, WI, USA) and examined for the presence of DNA inserts by EcoRI digestion.

### 2.6. DNA Sequencing

Plasmid DNA containing genomic DNA inserts was subjected to PCR using 7.5 × 10^−8^ M of M13 Forward (−29)/IRD700 and M13 Reverse/IRD800 dye-labeled primers (LI-COR, Lincoln, NE, USA) following the SequiTherm EXCEL II DNA Sequencing Kit-LC protocol for 66 cm gels (Epicentre Technologies, Madison, WI, USA). PCR products were electrophoresed in 3.6% polyacrylamide gel in TBE (Tris-Base EDTA) buffer on a LI-COR LongReadIR 4200 DNA Sequencer at 2000 V, 25.0 mA, and 45.0 W, at 45°C. Each DNA sequence was confirmed, in triplicate tests, by purifying and sequencing DNA from different clones. The nucleotide sequences were analyzed using the Vector NTI Suite program (InforMax, Inc., North Bethesda, MD, USA), a registered trademark of Life Technologies (Grand Island, NY, USA). The nucleotide sequences were aligned to known sequences using the BLAST program in the FASTA format.

### 2.7. Slot Blot Assay of Rattlesnake Venom

In brief, 2 *μ*L of rattlesnake venom was spotted onto nitrocellulose strips, washed five times in PBS, blocked in 5% goat serum, and incubated with metalloproteinase-specific antibodies AF5 and CAP-8, antibodies raised against anticomplement factors, and hemorrhagic metalloproteinases in *C. s. scutulatus* and *C. atrox*, respectively. The nitrocellulose strips were then washed extensively in PBS and incubated with secondary antibodies conjugated to horseradish peroxidase and developed using standard chromogenic reactions as previously described [3]. CAP-8 (a gift from Dr. John Perez, Texas A&M University, College Station, TX, USA) is a mouse monoclonal antibody that recognizes hemorrhagic rattlesnake venom proteins from rattlesnake venoms of *C. atrox*. AF5 is a polyclonal antibody raised against hemorrhagic and complement inactivating metalloproteinases derived from *C. s. scutulatus* venom. Antibody specificities have been characterized in previous studies [23–25].

### 2.8. Fibrinogenolytic Activity

Fibrinogenolytic activity in rattlesnake venom was assessed by mixing 160 *μ*L of a 1 mg/mL solution of human fibrinogen (93% clottable protein), dissolved in 0.03 M ammonium acetate (pH 7.5), with 16 *μ*L of rattlesnake venom (1 mg/mL) and 224 *μ*L of distilled water. Reaction mixtures were incubated at 37°C for 30 min. The reaction was terminated by adding 5 *μ*L of 0.5 M EDTA to 5 *μ*L aliquots of each sample. 

For some experiments, the biochemical activity associated with Zn^2+^-dependent metalloproteinases was assessed by incubating the reaction mixtures with EDTA to chelate zinc. In brief, 50 *μ*L of rattlesnake venom (1 mg/mL) was incubated with 20 mM EDTA for 1 hr at 37°C. The EDTA concentration in this mixture was two times higher in order to ensure the sequestration of Zn^2+^ and inactivation of metalloproteinases.

### 2.9. Fibrinolysis

Fibrinolytic activity was measured in fibrin-agarose plates that were prepared by mixing 2.5 mL of 1% agar with a 1% fibrinogen suspension to 35 mm Petri dishes containing 0.1 mL thrombin (0.5 units) to induce fibrin formation. Ten *μ*L of venom (1 mg/mL) was added to 3 mm wells in the agarose gels. Following incubation for 12 hr at 37°C in a humidified chamber, the diameters of the lysis zones induced by rattlesnake venom were measured with a ruler. In EDTA inhibition reactions, samples of venom were preincubated with 20 mM EDTA for 1 hr at 37°C. Standard deviations were determined from three separate repetitions of the procedure.

### 2.10. Caseinolysis

Caseinolytic activity was assayed in casein agar plates that were prepared by mixing agar with Protease Substrate Gel Tablets (Bio-Rad, Richmond, CA, USA) according to the manufacturer's protocol. Ten *μ*L of each sample (1 mg/mL venom in 0.1 M Tris-HCl buffer, pH 8.2) was dispersed into 4 mm diameter wells that were punched in the gel following polymerization of agar containing casein. The gels were then incubated at 37°C in humidified chamber for 12 hr. The caseinolytic activity of rattlesnake venom samples was determined by measuring the diameter of the hydrolysis zones. For EDTA treated reactions, venom samples were preincubated with 20 mM EDTA for 1 hr at 37°C. 

### 2.11. Esterase Activity

 Esterase activity was determined by adding 50 *μ*L of venom (1 mg/mL) to 300 *μ*L of either 0.01 M N-a-p-tosyl-L-arginine methyl ester (TAME) or 0.01 M N-a-bezoyl-L-arginine ethyl ester (BAEE) dissolved in 2.55 mL of 0.1 M Tris-HCl buffer, pH 8.2, containing 0.05 M CaCl_2_ and incubating for 1 hr at 37°C. Reaction was stopped by addition of 0.1 mL glacial acetic acid. Absorbance was read at 247 nm for TAME and 253 nm for BAEE. The activity is reported as the change in absorbance per min per mg protein. Each reaction was done at least in triplicate.

### 2.12. Hide Powder Azure Hydrolysis

Hydrolysis of hide powder azure was determined by adding 50 *µ*L of venom (1 mg/mL) to 5 mg of Remazol Brilliant Blue hide powder azure (Calbiochem Corp., La Jolla, CA, USA) suspended in 1.85 mL of 0.1 M Tris-HCl, 0.05 M CaCl_2_, pH 8.2. Reaction was terminated, after an incubation period of 2.5 hr at 37°C, by addition of 0.1 mL glacial acetic acid. The samples were centrifuged in a Microfuge for 15 min. For EDTA treated reactions, venom samples were preincubated with 20 mM EDTA for 1 hr at 37°C. Optical absorbance of the supernatant was determined at 595 nm. Specific activity is reported as the change in optical absorbance per min per mg protein. Each reaction was done at least in triplicate in three independent experiments.

### 2.13. Hemorrhagic Activity

Hemorrhagic activity of each rattlesnake venom sample was determined by injecting adult BALB/c mice subcutaneously in the dorsal region with 100 *μ*g of venom dissolved in 100 *μ*L of 0.85% NaCl. Following cervical dislocation, mice were examined for hemorrhage 4 hr after injection. Hemorrhagic activity is reported on a relative scale from zero to four, where four indicates high hemorrhagic activity (hemorrhagic area greater than 10 mm in diameter) and zero indicates no hemorrhagic activity.

## 3. Results

The enzymatic and hemorrhagic activities of fourteen *C. s. scutulatus* venoms are shown in Table 3. For comparative purposes, we also included data obtained from venoms from *C. atrox* (Call-1) and *C. m. molossus* (Cmm88) as positive controls for both proteolytic and hemorrhagic activities [26, 27]. We found that *C. s. scutulatus* venoms from Css28, Css31, and Css36 (collected from El Paso, TX, USA) showed potent hemorrhagic activity. Venom from three other *C. s. scutulatus* Css68, Css71, and Css74 collected in Maricopa County, AZ, USA, also induced hemorrhage to the same extent as venom derived from *C. atrox*. The other eight *C. s. scutulatus* venoms (collected from various geographic locations including Maricopa County, AZ, USA) did not cause hemorrhage (Table 3).

The presence of hemorrhagic toxins in venoms was also assessed by immunoblotting for hemorrhagic metalloproteinases (Table 3) using the slot blot method as previously published [3, 28]. Both AF5 and CAP8 antibodies immunoreacted with venoms that contained high hemorrhagic activity, whereas CAP8, but not AF5, recognized venoms with weak hemorrhagic activity (Css71 and Css74).

Significant differences were observed in proteolytic activities of rattlesnake venoms, particularly in the ability to hydrolyze fibrin, casein, and hide powder azure (HPA). A strong correlation was observed in the ability of rattlesnake venom to induce hemorrhage with its ability to hydrolyze HPA, an observation previously reported [16]. However, while all venoms were capable of hydrolyzing fibrinogen, some venoms (Css61, Css62, and Css64) lacked caseinolytic, fibrinolytic, and hemorrhagic activities (Table 3).

The venoms were pretreated with EDTA to ensure that the proteolytic activities were associated with metalloproteinases. The venoms were then tested for their ability to hydrolyze the esterase substrates TAME and BAEE. As shown in Table 3, all rattlesnake venoms had activity for both TAME and BAEE. The ability of EDTA pretreated venom to cleave fibrinogen varied (Table 4). While EDTA completely inhibited the fibrinogenolytic activity of venom Css61, EDTA was only able to partially inhibit fibrinogenolytic activity in four other venoms while having no effect on the rest of the rattlesnake venoms. These observations show that nonmetalloproteinase enzymes like esterase or serine proteases [29, 30] are capable of cleaving fibrinogen and were present in most of the venoms. However, hydrolysis of casein, fibrin, and HPA was completely inhibited by EDTA suggesting that all of the venoms contained zinc-dependent metalloproteinases that hydrolyze casein and fibrin, including venoms that lacked hemorrhagic activity and those that were not recognized by either the AF5 or CAP8 antibodies. In summary, all the biochemical activities including caseinolysis, fibrinolysis, and cleavage of HPA were attributed to metalloproteinases (Table 4).

Based on the results shown in Table 3, we were able to classify the snake venoms into four groups. Venoms from Css28, Css31, Css36, and Css68 were categorized in group one (GP1) based on their high proteolytic activity on all substrates analyzed and their high hemorrhagic activity. The second group (GP2) included Css61, Css62, and Css64. These three venoms hydrolyzed only fibrinogen but were not active against fibrin, casein, or hide powder azure and lacked hemorrhagic activity. The third group (GP3) included Css65, Css66, Css67, Css69, and Css75, all of which were active on all protein substrates except high powder azure and did not cause hemorrhage. Finally, the fourth group (GP4) included Css71 and Css74. These venoms were active on all protein substrates but were weakly hemorrhagic (Table 3).

**Figure 1:**
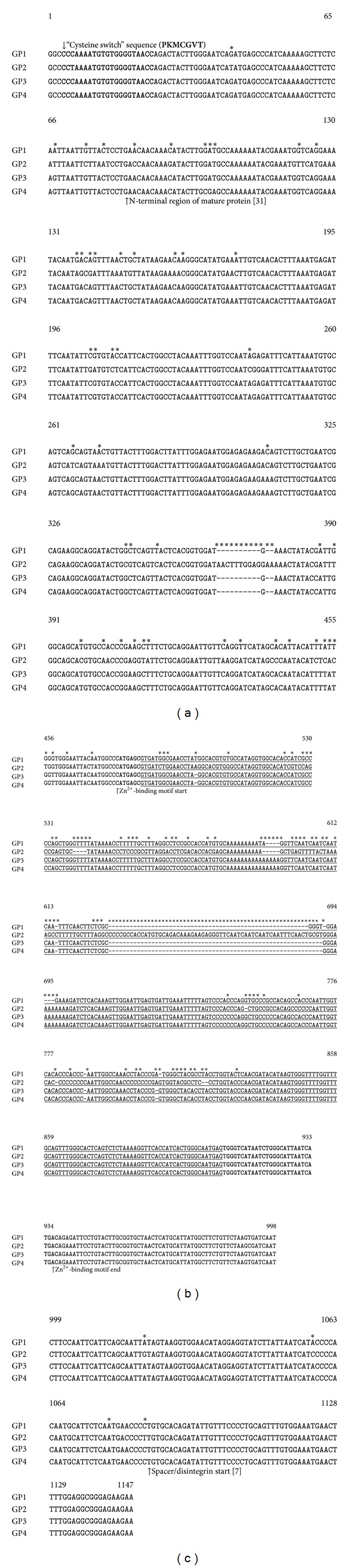
Alignment of the four groups (GP1, GP2, GP3, and GP4) of *C. s. scutulatus* metalloproteinase genomic DNA sequences. Assignment of exon and intron (underlined) regions was made by comparing metalloproteinase cDNA sequences from *C. atrox* [7] and *A. contortrix *[12, 30] with metalloproteinase genomic DNA sequences from *C. s. scutulatus* obtained in this study. Differences among the DNA sequences are denoted by an asterisk (*).

Genomic DNA sequences (four groups of genomic metalloproteinase DNA sequences, GP-I, GP-II, GP-III, and GP-IV, have been deposited in the GenBank under the accession numbers AF378673, AF378674, AF378675, and AF378676, resp.) were subsequently determined for the metalloproteinase genes. Using primers shown in Table 2, we were able to PCR-amplify 1,050 base pair metalloproteinase specific gene fragments for each of the 14 *C. s. scutulatus* rattlesnakes. The PCR-amplified nucleotide fragments were extracted from the agarose gels, subcloned into TOPO vectors, and sequenced (Figure 1). Following DNA sequence alignments using BLAST algorithm, each genomic DNA sample showed high homology to known metalloproteinase genes. The highest sequence identity belonged to the cDNAs of *C. atrox* Atrolysins [7] and *Agkistrodon contortrix* (copperhead) metalloproteinase genes [11, 31–33]. One intron was identified to be present in all of the partial genomic metalloproteinase DNA sequences analyzed in the sample *C. s. scutulatus* population. This intron was located seven nucleotides (from the 5′ end) inside the Zn^2+^-binding site (base pair number 476, see Supplementary Figure 1 in supplementary material available online at http://dx.doi.org/10.1155/2013/251474). This intron had a classical consensus splice-site (GT donor and AG acceptor) sequence (Supplementary Figure 1).

All of the nucleotide sequences contained similar structural domains including a conserved “cysteine switch” sequence [34] located within the zymogen region, an N-terminal region of the proteinase domain, a Zn^2+^-binding domain, and a spacer domain located between the proteinase and disintegrin domains. The homology among the genomic DNA metalloproteinase sequences was greater than 95% for the zymogen region (nucleotides 1–82), 100% for the zinc-binding domain (Table 5), and greater than 98% for the spacer region (nucleotides 1084 to 1147). The DNA sequence homology for the proteinase domain across all 14 *C. s. scutulatus* genomic metalloproteinase genes, excluding the intron (nucleotides 83 to 474 and 909 to 1083), was close to 89%, and the sequence identity for the entire gene was at least 84.5% homologous among the four groups of metalloproteinase genes (Table 5). However, the sequence homology within the intron region among the fourteen snakes (nucleotides 476 to 908) was highly variable (~67% homology for some rattlesnakes, Table 5) suggesting that differences in the splicing of introns and fusion of exons among the four different groups of rattlesnake venoms may contribute to variations in metalloproteinase activities as shown in Table 3.

A thorough analysis of the DNA nucleotide sequence showed that the fourteen metalloproteinase genomic DNA sequences exhibited a perfect correlation with the biochemical classification of the rattlesnake venoms. The genomic DNA sequences from Css28, Css31, Css36, and Css68 were classified in group one (GP1), whereas genomic DNA sequences belonging to Css61, Css62, and Css64 were classified into group two (GP2). The genomic DNA sequences belonging to Css65, Css66, Css67, Css69, and Css75 were categorized into group three (GP3), whereas genomic DNA sequences belonging to Css71 and Css74 were classified in group four (GP4) (Figure 1). This classification is identical to the classification that was based on the biochemical profiles of the fourteen rattlesnake venoms pointing to the existence of a genetic basis that gives rise to intra-species variation in metalloproteinase-associated activities in *C. s. scutulatus*. Interestingly, the genomic DNA sequence belonging to GP2 differed the most compared to the other three groups in that it contained a longer intron sequence (Table 5).

## 4. Discussion

*C. s. scutulatus* is an excellent rattlesnake species for studying intra-species variations in rattlesnake venom due to its high variation in metalloproteinase composition and biochemical activities. Previous published work has shown that an inverse relationship exists between the presence of the Mojave toxin (a potent neurotoxin) and the presence of hemorrhagic toxins (metalloproteinase) in the venom of this species of rattlesnakes [15, 16]. Our biochemical data confirm our previous observations that the Mojave rattlesnake venom is highly variable across different geographic locations. In the sample population analyzed for this study, we found that the rattlesnake venom derived from a mother and two of her offspring contained strong hemorrhagic activity. These rattlesnakes were captured in El Paso County, TX, USA. Moreover, a rattlesnake containing strong hemorrhagic activity was collected in Maricopa County, AZ, USA. Two other snakes that were collected in Maricopa County, AZ, USA, also had modest hemorrhagic activity, whereas other snakes caught in Texas, New Mexico, and some in Arizona had venoms with weak proteolytic activity and no hemorrhagic activity. Overall, these observations attest to the importance of producing antisera against biochemically different types of Mojave rattlesnake venom.

Rattlesnakes have different types of metalloproteinases in their venom, which have been classified according to their hemorrhagic, fibrinogenolytic and/or fibrinolytic, anticomplement, and a combination of these proteolytic activities [5, 6, 23, 35, 36]. Fibrinogen, fibrin, casein, and hide powder azure are substrates often used to measure proteolytic activity of crude rattlesnake venom or components of venom. A broad-spectrum proteolytic activity against different substrates is associated with high hemorrhagic activity. In the present study, we assessed metalloproteinase activities in Mojave rattlesnake venom based on its ability to hydrolyze different substrates. We found significant intra-species differences in venom metalloproteinase-related biochemical activity and hemorrhagic activity for *C. s. scutulatus* rattlesnake. Mojave rattlesnake venoms that were active against hide powder azure correlated with high hemorrhagic activity and had broad activity against all other substrates. Rattlesnake venoms that were active against three of four substrates had modest to weak hemorrhagic activity suggesting that a combination of proteolytic and fibrinogenolytic enzymes are required to induce potent hemorrhagic activity (Table 3). Pretreatment with EDTA, as expected, resulted in a complete loss of metalloproteinase-specific activities, except for fibrinogen, which was hydrolyzed by all rattlesnake venoms even in the presence of EDTA suggesting that all *C. s. scutulatus* venoms contained esterase and/or serine proteases, enzymes known to hydrolyze fibrinogen in the absence of metals [29, 30]. 

Other species of the Crotalidae family also show strong intra-species variation. Indeed, we have previously shown that a population of *C. m. molossus* had up to eleven fibrin hydrolyzing enzymes [4, 5] and at least 13 metalloproteinases including Ht-a, Ht-b, Ht-c, Ht-d, Ht-e, Ht-f, Ht-g, VAP1, VAP2, Catroxase, and Atroxases [36]. Unlike all the other comparative biochemical studies previously done, this is the first report to show a genetic basis for differences in metalloproteinase activity of the Mojave rattlesnake. Prior to this study, the molecular mechanisms that give rise to the variability in rattlesnake venom metalloproteinase activity within members of the same rattlesnake species such as *C. scutulatus scutulatus* were not known. Other investigators have postulated that certain environmental factors and/or diet associated with geographical location may modulate the transcription, translation, and/or posttranslational processing of metalloproteinases and contribute to intraspecies variation of rattlesnake biochemical profiles. 

Furthermore, this is the first study to categorize Mojave rattlesnake metalloproteinases into four groups based on their DNA homology and biochemical profiles. Indeed, Groups 2, 3, and 4 Mojave rattlesnake metalloproteinase genes have few single nucleotide differences that may encode for minor coding differences at the N-terminal and central regions of the proteinase domain that may likely decrease or nullify the hemorrhagic activities of these three groups of metalloproteinase genes. Interestingly, the C-terminal regions of the metalloproteinase genes are very well conserved among the four groups, which suggests that the presence of additional secondary structural elements contributes to hemorrhagic induction and that they are distinct from the disintegrin domain, a region involved in inhibiting platelet aggregation. Although both subclasses of metalloproteinases may show proteolytic activity towards different substrates, hemorrhagic activity may be imparted by additional structural domains beyond the proteinase domain. Based on the biochemical profile and genomic DNA sequencing data, we believe that Group 1 of Mojave rattlesnake metalloproteinase genes is classified as a P-1A metalloproteinase, whereas other groups are likely classified as P-1B metalloproteinase given their lack or little hemorrhagic activity. Interestingly, none of the groups correlated with geographic location but rather showed a perfect association with their biochemical profiles suggesting that each group displays different patterns of migration across the southwest region of the USA. Interestingly, our results suggest that single nucleotide polymorphisms within the mature proteinase domain may further contribute to secondary structural differences that lead to differences in catalytic activity of metalloproteinases. 

In this study, we discovered the presence of an intron that is located within the metal-binding region of the metalloproteinase sequence. Alternative splicing of a large primary transcript has also been proposed as an alternative molecular mechanism that gives rise to differences in metalloproteinase composition and biochemical profiles of rattlesnake venom [7]. This model suggests that a single large primary transcript gives rise to P-I, P-II, and/or P-III metalloproteinases by alternative splicing of a large metalloproteinase primary transcript. The observation in this study that the noncoding regions for the four groups of rattlesnakes showed low homology (~67%) compared to the exon coding regions suggests that possible alternate splicing mechanisms can give rise to different metalloproteinase splice variants leading to differences in metalloproteinase composition of rattlesnake venom of *C. s. scutulatus*. Indeed, the intron in GP2 was the least similar to the other three groups. Interestingly, while all genomic DNA sequences contained the same consensus splice sites, the DNA homology of the intron region was at least 67% between GP2 and the other groups of the metalloproteinase genes sequenced in *C. s. scutulatus* rattlesnakes. Although there is a general consensus that exons are more conserved than introns within the genes of the same family [37], a 67% nucleotide sequence identity for the intron regions especially for rattlesnakes belonging to the same species is quite low and unexpected. 

The untranslated region sequences (UTR) in mRNA transcripts are small intronic regions contained in genes [37] that produce secondary RNA structures involved in the regulation of translation [38]. In this study, we demonstrated that the intron regions of *C. s. scutulatus* contain an unusually low DNA sequence homology, which gives rise to the possibility that different secondary structures generated by introns may modulate transcription of metalloproteinases. Overall, based on our genomic DNA sequences and biochemical data, we believe that a combination of alternative splicing, single nucleotide polymorphisms within the catalytic regions of the proteinase domains, and differences in UTR structures contribute to intra-species variation in metalloproteinase composition and hemorrhagic activity in the Mojave rattlesnake. However, we do recognize that our results do not directly show that alternative splicing contributes to differences in rattlesnake venom composition and metalloproteinase-associated activities, and future *in vitro* transcription/translation studies are required to conclusively test this hypothesis.

## 5. Conclusions

We identified four groups of *C. s. scutulatus* rattlesnake venoms with each having distinct biochemical profiles associated with metalloproteinase activity. Rattlesnakes from each venom group had unique genomic nucleotide DNA sequences for the mature metalloproteinase domain and noncoding regions. These findings support the genomic basis underlying diversity in venom metalloproteinase activities. This is the first report on genomic DNA sequences of snake venom metalloproteinases.

## Supplementary Material

Supplementary Figure 1. DNA alignment of fourteen *C. s. scutulatus* metalloproteinase genomic DNA sequences.
The assignment of exon and intron (underlined) regions were identified by comparing the metalloproteinase cDNA sequences from *C. atrox* [7] and *A. contortrix* [11, 31-33] with the *C. s. scutulatus* metalloproteinase genomic DNA sequences obtained in this study. Structural domains are indicated in bold. Nucleotide differences among the genomic DNA sequences are denoted by an asterisk (∗).

## Figures and Tables

**Table 1:** Geographical data of Mojave rattlesnakes captured for this study.

Snake	Locality
Css28	El Paso County, TX, USA
Css31	Offspring of Css28
Css36	Offspring of Css28
Css61	Hudspeth County, TX, USA
Css62	Hudspeth County, TX, USA
Css64	Hidalgo County, NM, USA
Css65	Cochise County, AZ, USA
Css66	Cochise County, AZ, USA
Css67	Maricopa County, AZ, USA
Css68	Maricopa County, AZ, USA
Css69	Maricopa County, AZ, USA
Css71	Maricopa County, AZ, USA
Css74	Maricopa County, AZ, USA
Css75	Maricopa County, AZ, USA

**Table 2:** Primers used for amplifying metalloproteinase sequences from *Crotalus s. scutulatus.* Genomic DNA and sequence comparison to other snake venom metalloproteinases (MP). Nonconserved nucleotides are indicated in bold.

Primer	Annealing site	Corresponding sequence
**MP (sense)**		GCCC**C**CAAAATGTGTGGGGTAAC
	Atrolysin*b* (558–580)	GCCC**C**CAAAATGTGTGGGGTAAC
	Artolysin*c* (567–589)	GCCC**C**CAAAATGTGTGGGGTAAC
	Atrolysin*d* (488–510)	GCCC**C**CAAAATGTGTGGGGTAAC
	Atrolysin*e* (548–570)	GCCC**C**CAAAATGTGTGGGGTAAC
	Catrocollastatin (537–559)	GCCC**T**CAAAATGTGTGGGGTAAC

**MP (antisense)**		TTCT**TC**TCC**CG**C**C**TCCAAAAGTTC
	Atrolysin*a* (1329–1306)	TTCT**TC**TCC**CA**C**C**TCCAAAAGTTC
	Atrolysin*b* (1328–1305)	TTCT**TC**TCC**CG**C**C**TCCAAAAGTTC
	Atrolysin*c* (1327–1304)	TTCT**TC**TCC**CG**C**C**TCCAAAAGTTC
	Atrolysin*d* (1248–1225)	TTCT**TC**TCC**CG**C**C**TCCAAAAGTTC
	Atrolysin*e* (1308–1285)	TTCT**AT**TCC**CG**C**C**TCCAAAAGTTC
	Catrocollastatin (1297–1274)	TTCT**TC**TCC**GG**C**T**TCCAAAAGTTC

**Table 3:** Antibody recognition: proteinase and hemorrhagic activities of the *C. s. scutulatus* venoms.

Snake	Fibrinogenolytic activity (peptide cleaved)	Fibrinolytic activity (mm)	Caseinolytic activity (mm)	TAME^a^ hydrolysis (units)	BAEE^b^ hydrolysis (units)	HP azure^c^ hydrolysis (units)	HEM^d^ activity (intensity)	AF5	CAP8
Css28	*αβ*	11 ± 0.3^e^	9.0 ± 0.3	1.66 ± 0.03	1.131 ± 0.02	0.161 ± 0.01	4+	+	+
Css31	*αβ*	8.0 ± 0.2	7.0 ± 0.2	1.40 ± 0.02	1.30 ± 0.02	0.211 ± 0.01	4+	+	+
Css36	*αβ*	6.0 ± 0.2	6.0 ± 0.2	1.15 ± 0.01	0.901 ± 0.01	0.171 ± 0.01	4+	+	+
Css61	*αβ*	0	0	1.72 ± 0.02	0.691 ± 0.05	0	0	−	−
Css62	*αβ*	0	0	0.18 ± 0.01	0.441 ± 0.03	0	0	−	−
Css64	*αβ*	0	0	0.931 ± 0.02	0.51 ± 0.02	0	0	−	−
Css65	*αβ*	15 ± 0.6	3.5 ± 0.1	3.50 ± 0.01	ND^f^	0	0	−	−
Css66	*αβ*	4.0 ± 0.1	6.01 ± 0.3	0.261 ± 0.02	0.481 ± 0.03	0	0	−	−
Css67	*αβ*	5.0 ± 0.2	5.0 ± 0.2	0.161 ± 0.01	0.061 ± 0.001	0	0	−	−
Css68	*αβ*	8.0 ± 0.3	11.0 ± 0.4	0.081 ± 0.001	0.081 ± 0.001	0.281 ± 0.01	4+	+	+
Css69	*αβ*	6.0 ± 0.2	4.0 ± 0.1	0.50 ± 0.01	ND	0	0	−	−
Css71	*αβ*	6.0 ± 0.2	3.5 ± 0.1	0.301 ± 0.01	0.441 ± 0.03	0.061 ± 0.02	1+	−	+
Css74	*αβ*	6.0 ± 0.2	6.5 ± 0.2	0.221 ± 0.02	0.221 ± 0.01	0.061 ± 0.01	1+	−	+
Css75	*αβ*	8.0 ± 0.3	3.51 ± 0.1	0.321 ± 0.03	0.321 ± 0.01	0	0	−	−
Call-1	*αβ*	18 ± 0.5	14.0 ± 0.5	ND	ND	0.5191 ± 0.02	4+	+	+
Cmm88	*αβ*	22 ± 0.9	16.0 ± 0.6	0.881 ± 0.214	0.871 ± 0.32	0.321 ± 0.01	4+	ND	ND

^a^N-a-Tosyl-L-arginine methyl ester.

^b^N-a-Benzoyl-L-arginine ethyl ester.

^c^Hide powder azure.

^d^Hemorrhagic.

^e^Mean ± the standard deviation based on three repetitions.

^f^No data.

**Table 4:** Proteolytic activity inhibition of the *C. s. scutulatus* venoms by EDTA.

Snake	Inhibition of fibrinogenolysis (peptide cleaved)	Inhibition of caseinolysis and fibrinolysis	Inhibition of hide powder azure hydrolysis
Css28	*β*	+^a^	+
Css31	*β*	+	+
Css36	*αβ*	+	+
Css61	None	NA^b^	NA
Css62	*β*	NA	NA
Css64	*β*	NA	NA
Css65	*αβ*	+	NA
Css66	*αβ*	+	NA
Css67	*αβ*	+	NA
Css68	*β*	+	+
Css69	*αβ*	+	NA
Css71	*αβ*	+	+
Css74	*αβ*	+	+
Css75	*αβ*	+	NA

^a^+ indicates that the activity was inhibited 100% by EDTA.

^b^Not applicable due to the lack of activity when not treated with EDTA (refer to Table 3).

**Table 5:** DNA sequence homology for different structural domains of the four groups of *C. s. scutulatus* metalloproteinase genes.

Group number domain	Total	Proteinase domain	Intron	Zinc-binding domain
GP1 versus GP2	84.5%	89%	67.3%	100%
GP1 versus GP3	98%	99%	94.7%	100%
GP1 versus GP4	98%	99%	96%	100%
GP2 versus GP3	83%	89%	67%	100%
GP2 versus GP4	83%	90%	70%	100%
GP3 versus GP4	99%	99%	100%	100%

## Abbreviations

PCR: Polymerase chain reaction
TAME: N-a-p-Tosyl-L-arginine methyl ester
BAEE: N-a-Benzoyl-L-arginine ethyl ester
EDTA: Ethylenediaminetetraacetic acid
cDNA: Complementary DNA
mRNA: Messenger RNA
UTR: Untranslated region.
