# Supplementary material for: Genetic Basis for Variation of Metalloproteinase-Associated Biochemical Activity in Venom of the Mojave Rattlesnake (Crotalus scutulatus scutulatus)
Source: Biochem Res Int. 2013 Jul 29;2013:251474. doi: 10.1155/2013/251474 (PMC3745941; doi:10.1155/2013/251474)
Supplement: Supplementary file 1 — Supplementary Figure 1. DNA alignment of fourteen C. s. scutulatus metalloproteinase genomic DNA sequences. The assignment of exon and intron (underlined) regions were identified by comparing the metalloproteinase cDNA sequences from C. atrox [7] and A. contortrix [11, 31-33] with the C. s. scutulatus metalloproteinase genomic DNA sequences obtained in this study. Structural domains are indicated in bold. Nucleotide differences among the genomic DNA sequences are denoted by an asterisk (∗). [file 251474.f1.pdf]

Dagda et al., 2013 Biochemistry Research International

Supplementary Figure 1

1 65
* ↓"Cysteine Switch" sequence **(PKMCGVT)** *****

Css28 GGC**CCCAAAATGTGTGGGGTAACC**AGACTACTTGGGAATCAGATGAGCCCATCAAAAAGCTTCTC
Css31 GGC**CCCAAAATGTGTGGGGTAACC**AGACTACTTGGGAATCAGATGAGCCCATCAAAAAGCTTCTC
Css36 GGC**CCCAAAATGTGTGGGGTAACC**AGACTACTTGGGAATCAGATGAGCCCATCAAAAAGCTTCTC
Css61 GCC**CCCAAAATGTGTGGGGTAACC**AGACTACTTGGGAATCATATGAGCCCATCAAAAAGCTTCTC
Css62 GCC**CCCAAAATGTGTGGGGTAACC**AGACTACTTGGGAATCATATGAGCCCATCAAAAAGCTTCTC
Css64 GCC**CCCAAAATGTGTGGGGTAACC**AGACTACTTGGGAATCATATGAGCCCATCAAAAAGCTTCTC
Css65 GCC**CCCAAAATGTGTGGGGTAACC**AGACTACTTGGGAATCAGATGAGCCCATCAAAAAGCTTCTC
Css66 GCC**CCCAAAATGTGTGGGGTAACC**AGACTACTTGGGAATCAGATGAGCCCATCAAAAAGCTTCTC
Css67 GCC**CCCAAAATGTGTGGGGTAACC**AGACTACTTGGGAATCAGATGAGCCCATCAAAAAGCTTCTC
Css68 GGC**CCCAAAATGTGTGGGGTAACC**AGACTACTTGGGAATCAGATGAGCCCATCAAAAAGCTTCTC
Css69 GCC**CCCAAAATGTGTGGGGTAACC**AGACTACTTGGGAATCAGATGAGCCCATCAAAAAGCTTCTC
Css71 GCC**CCCAAAATGTGTGGGGTAACC**AGACTACTTGGGAATCAAATGAGCCCATCAAAAAGCTTCTC
Css74 GCC**CCCAAAATGTGTGGGGTAACC**AGACTACTTGGGAATCAAATGAGCCCATCAAAAAGCTTCTC
Css75 GCC**CCCAAAATGTGTGGGGTAACC**AGACTACTTGGGAATCAGATGAGCCCATCAAAAAGCTTCTC

66 130
* * * * * *** * *

Css28 AATTAATTGTTACTCCTGAACAACAAACATACTTGGATGCCAAAAAAATACGAAATGGTCAGGAAA
Css31 AATTAATTGTTACTCCTGAACAACAAACATACTTGGATGCCAAAAAAATACGAAATGGTCAGGAAA
Css36 AATTAATTGTTACTCCTGAACAACAAACATACTTGGATGCCAAAAAAATACGAAATGGTCAGGAAA
Css61 ATTTAATTCTTAATCCTGACCAACAAAGATACTTGGATGCCAAAAAAATACGAAATGTTCATGAAA
Css62 ATTTAATTCTTAATCCTGACCAACAAAGATACTTGGATGCCAAAAAAATACGAAATGTTCATGAAA
Css64 ATTTAATTCTTAATCCTGACCAACAAAGATACTTGGATGCCAAAAAAATACGAAATGTTCATGAAA
Css65 AGTTAATTGTTACTCCTGAACAACAAACATACTTGGATGCCAAAAAAATACGAAATGGTCAGGAAA
Css66 AGTTAATTGTTACTCCTGAACAACAAACATACTTGGATGCCAAAAAAATACGAAATGGTCAGGAAA
Css67 AGTTAATTGTTACTCCTGAACAACAAACATACTTGGATGCCAAAAAAATACGAAATGGTCAGGAAA
Css68 AATTAATTGTTACTCCTGAACAACAAACATACTTGGATGCCAAAAAAATACGAAATGGTCAGGAAA
Css69 AGTTAATTGTTACTCCTGAACAACAAACATACTTGGATGCCAAAAAAATACGAAATGGTCAGGAAA
Css71 AGTTAATTGTTACTCCTGAACAACAAACATACTTGCGAGCCAAAAAAATACGAAATGGTCAGGAAA
Css74 AGTTAATTGTTACTCCTGAACAACAAACATACTTGCGAGCCAAAAAAATACGAAATGGTCAGGAAA
Css75 AGTTAATTGTTACTCCTGAACAACAAACATACTTGGATGCCAAAAAAATACGAAATGGTCAGGAAA
↑ N-terminal region of mature protein (de Araujo and Ownby, 1995)

131 195
** ** * * * * *

Css28 TACAATGACAGTTTAACTGCTATAAGAACAAGGGCATATGAAATTGTCAACACTTTAAATGAGAT
Css31 TACAATGACAGTTTAACTGCTATAAGAACAAGGGCATATGAAATTGTCAACACTTTAAATGAGAT
Css36 TACAATGACAGTTTAACTGCTATAAGAACAAGGGCATATGAAATTGTCAACACTTTAAATGAGAT
Css61 TACAATAGCGATTTAAATGTTATAAGAAAACGGGCATATGAACTTGTCAACACTTTAAATGAGAT
Css62 TACAATAGCGATTTAAATGTTATAAGAAAACGGGCATATGAACTTGTCAACACTTTAAATGAGAT
Css64 TACAATAGCGATTTAAATGTTATAAGAAAACGGGCATATGAACTTGTCAACACTTTAAATGAGAT
Css65 TACAATGACAGTTTAACTGCTATAAGAACAAGGGCATATGAAATTGTCAACACTTTAAATGAGAT
Css66 TACAATGACAGTTTAACTGCTATAAGAACAAGGGCATATGAAATTGTCAACACTTTAAATGAGAT
Css67 TACAATGACAGTTTAACTGCTATAAGAACAAGGGCATATGAAATTGTCAACACTTTAAATGAGAT
Css68 TACAATGACAGTTTAACTGCTATAAGAACAAGGGCATATGAAATTGTCAACACTTTAAATGAGAT
Css69 TACAATGACAGTTTAACTGCTATAAGAACAAGGGCATATGAAATTGTCAACACTTTAAATGAGAT
Css71 TACAATGACAGTTTAACTGCTATAAGAACAAGGGCATATGAAATTGTCAACACTTTAAATGAGAT
Css74 TACAATGACAGTTTAACTGCTATAAGAACAAGGGCATATGAAATTGTCAACACTTTAAATGAGAT
Css75 TACAATGACAGTTTAACTGCTATAAGAACAAGGGCATATGAAATTGTCAACACTTTAAATGAGAT

```
           196                                                           260
                    **   **                              *
Css28      TTCAATATTCGTGTACCATTCACTGGCCTACAAATTTGGTCCAATAGAGATTTCATTAAATGTGC
Css31      TTCAATATTCGTGTACCATTCACTGGCCTACAAATTTGGTCCAATAGAGATTTCATTAAATGTGC
Css36      TTCAATATTCGTGTACCATTCACTGGCCTACAAATTTGGTCCAATAGAGATTTCATTAAATGTGC
Css61      TTCAATATTGATGTCTCATTCACTGGCCTACAAATTTGGTCCAATACAGATTTCATTAAATGTGC
Css62      TTCAATATTGATGTCTCATTCACTGGCCTACAAATTTGGTCCAATACAGATTTCATTAAATGTGC
Css64      TTCAATATTGATGTCTCATTCACTGGCCTACAAATTTGGTCCAATACAGATTTCATTAAATGTGC
Css65      TTCAATATTCGTGTACCATTCACTGGCCTACAAATTTGGTCCAATAGAGATTTCATTAAATGTGC
Css66      TTCAATATTCGTGTACCATTCACTGGCCTACAAATTTGGTCCAATAGAGATTTCATTAAATGTGC
Css67      TTCAATATTCGTGTACCATTCACTGGCCTACAAATTTGGTCCAATAGAGATTTCATTAAATGTGC
Css68      TTCAATATTCGTGTACCATTCACTGGCCTACAAATTTGGTCCAATAGAGATTTCATTAAATGTGC
Css69      TTCAATATTCGTGTACCATTCACTGGCCTACAAATTTGGTCCAATAGAGATTTCATTAAATGTGC
Css71      TTCAATATTCGTGTACCATTCACTGGCCTACAAATTTGGTCCAATAGAGATTTCATTAAATGTGC
Css74      TTCAATATTCGTGTACCATTCACTGGCCTACAAATTTGGTCCAATAGAGATTTCATTAAATGTGC
Css75      TTCAATATTCGTGTACCATTCACTGGCCTACAAATTTGGTCCAATAGAGATTTCATTAAATGTGC

           261                                                           325
                       *                                    *
Css28      AGTCAGCAGTAACTGTTACTTTGGACTTATTTGGAGAATGGAGAGAAGACAGTCTTGTTGAATCG
Css31      AGTCAGCAGTAACTGTTACTTTGGACTTATTTGGAGAATGGAGAGAAGACAGTCTTGTTGAATCG
Css36      AGTCAGCAGTAACTGTTACTTTGGACTTATTTGGAGAATGGAGAGAAGACAGTCTTGTTGAATCG
Css61      AGTCAGTAGTAAATGTTACTTTGGACTTATTTGGAGAATGGAGAGAAGACAGTCTTGTTGAATCG
Css62      AGTCAGTAGTAAATGTTACTTTGGACTTATTTGGAGAATGGAGAGAAGACAGTCTTGTTGAATCG
Css64      AGTCAGTAGTAAATGTTACTTTGGACTTATTTGGAGAATGGAGAGAAGACAGTCTTGTTGAATCG
Css65      AGTCAGTAGTAACTGTTACTTTGGACTTATTTGGAGAATGGAGAGAAGAAAGTCTTGTTGAATCG
Css66      AGTCAGTAGTAACTGTTACTTTGGACTTATTTGGAGAATGGAGAGAAGAAAGTCTTGTTGAATCG
Css67      AGTCAGTAGTAACTGTTACTTTGGACTTATTTGGAGAATGGAGAGAAGAAAGTCTTGTTGAATCG
Css68      AGTCAGCAGTAACTGTTACTTTGGACTTATTTGGAGAATGGAGAGAAGACAGTCTTGTTGAATCG
Css69      AGTCAGTAGTAACTGTTACTTTGGACTTATTTGGAGAATGGAGAGAAGAAAGTCTTGTTGAATCG
Css71      AGTCAGTAGTAACTGTTACTTTGGACTTATTTGGAGAATGGAGAGAAGAAAGTCTTGTTGAATCG
Css74      AGTCAGTAGTAACTGTTACTTTGGACTTATTTGGAGAATGGAGAGAAGAAAGTCTTGTTGAATCG
Css75      AGTCAGTAGTAACTGTTACTTTGGACTTATTTGGAGAATGGAGAGAAGAAAGTCTTGTTGAATCG

           326                                                           390
                           **      *             ********** **         *   *
Css28      CAGAAGGCAGGATACTGGCTCAGTTACTCACGGTGGAT----------G--AAACTATACGATTG
Css31      CAGAAGGCAGGATACTGGCTCAGTTACTCACGGTGGAT----------G--AAACTATACGATTG
Css36      CAGAAGGCAGGATACTGGCTCAGTTACTCACGGTGGAT----------G--AAACTATACGATTG
Css61      CAGAAGGCAGGATACTGCGTCAGTCACTCACGGTGGATAACTTTGGAGGAAAAACTATACGATTT
Css62      CAGAAGGCAGGATACTGCGTCAGTCACTCACGGTGGATAACTTTGGAGGAAAAACTATACGATTT
Css64      CAGAAGGCAGGATACTGCGTCAGTCACTCACGGTGGATAACTTTGGAGGAAAAACTATACGATTT
Css65      CAGAAGGCAGGATACTGGCTCAGTTACTCACGGTGGAT----------G--AAACTATACCATTG
Css66      CAGAAGGCAGGATACTGGCTCAGTTACTCACGGTGGAT----------G--AAACTATACCATTG
Css67      CAGAAGGCAGGATACTGGCTCAGTTACTCACGGTGGAT----------G--AAACTATACCATTG
Css68      CAGAAGGCAGGATACTGGCTCAGTTACTCACGGTGGAT----------G--AAACTATACGATTG
Css69      CAGAAGGCAGGATACTGGCTCAGTTACTCACGGTGGAT----------G--AAACTATACCATTG
Css71      CAGAAGGCAGGATACTGGCTCAGTTACTCACGGTGGAT----------G--AAACTATACCATTG
Css74      CAGAAGGCAGGATACTGGCTCAGTTACTCACGGTGGAT----------G--AAACTATACCATTG
Css75      CAGAAGGCAGGATACTGGCTCAGTTACTCACGGTGGAT----------G--AAACTATACCATTG

           391                                                           455
                  *     *  *   * **                 * *       *  *     * ***
Css28      GGCAGCATGTGCCACCCGAAGCTTTCTGCAGGAATTGTTCAGGTTCATAGCACATTACATTTATT
```

```
Css31   GGCAGCATGTGCCACCCGAAGCTTTCTGCAGGAATTGTTCAGGTTCATAGCACATTACATTTATT
Css36   GGCAGCATGTGCCACCCGAAGCTTTCTGCAGGAATTGTTCAGGTTCATAGCACATTACATTTATT
Css61   GGCAGCACGTGCAACCCGAGGTATTCTGCAGGAATTGTTAAGGATCATAGCCCAATACATCTCAC
Css62   GGCAGCACGTGCAACCCGAGGTATTCTGCAGGAATTGTTAAGGATCATAGCCCAATACATCTCAC
Css64   GGCAGCACGTGCAACCCGAGGTATTCTGCAGGAATTGTTAAGGATCATAGCCCAATACATCTCAC
Css65   GGCAGCATGTGCCACCGGAAGCTTTCTGCAGGAATTGTTCAGGATCATAGCACAATACATTTTAT
Css66   GGCAGCATGTGCCACCGGAAGCTTTCTGCAGGAATTGTTCAGGATCATAGCACAATACATTTTAT
Css67   GGCAGCATGTGCCACCGGAAGCTTTCTGCAGGAATTGTTCAGGATCATAGCACAATACATTTTAT
Css68   GGCAGCATGTGCCACCCGAAGCTTTCTGCAGGAATTGTTCAGGTTCATAGCACATTACATTTATT
Css69   GGCAGCATGTGCCACCGGAAGCTTTCTGCAGGAATTGTTCAGGATCATAGCACAATACATTTTAT
Css71   GGCAGCATGTGCCACCGGAAGCTTTCTGCAGGAATTGTTCAGGATCATAGCACAATACATTTTAT
Css74   GGCAGCATGTGCCACCGGAAGCTTTCTGCAGGAATTGTTCAGGATCATAGCACAATACATTTTAT
Css75   GGCAGCATGTGCCACCGGAAGCTTTCTGCAGGAATTGTTCAGGATCATAGCACAATACATTTTAT

        456                                                                   530
        *  *    *       *                   ***      *      *               * * ***
Css28   GGGTGGGAATTACAATGGCCCATGAGCGTGATGGCGAACCTATGGCACGTGTGCCATAGGTGGCACACCATCGCC
Css31   GGGTGGGAATTACAATGGCCCATGAGCGTGATGGCGAACCTATGGCACGTGTGCCATAGGTGGCACACCATCGCC
Css36   GGGTGGGAATTACAATGGCCCATGAGCGTGATGGCGAACCTATGGCACGTGTGCCATAGGTGGCACACCATCGCC
Css61   TGGTGGGAATTACTATGGCCCATGAGCGTGATCTGGAACCTAAGGCACGGGTGCCATAGGTGGCACATCGTCCAG
Css62   TGGTGGGAATTACTATGGCCCATGAGCGTGATCTGGAACCTAAGGCACGGGTGCCATAGGTGGCACATCGTCCAG
Css64   TGGTGGGAATTACTATGGCCCATGAGCGTGATCTGGAACCTAAGGCACGGGTGCCATAGGTGGCACATCGTCCAG
Css65   GGTTGGAAATTACAATGGCCCATGAGCGTGATGGCGAACCTA-GGCACGTGTGCCATAGGTGGCACACCATCGCC
Css66   GGTTGGAAATTACAATGGCCCATGAGCGTGATGGCGAACCTA-GGCACGTGTGCCATAGGTGGCACACCATCGCC
Css67   GGTTGGAAATTACAATGGCCCATGAGCGTGATGGCGAACCTA-GGCACGTGTGCCATAGGTGGCACACCATCGCC
Css68   GGGTGGGAATTACAATGGCCCATGAGCGTGATGGCGAACCTATGGCACGTGTGCCATAGGTGGCACACCATCGCC
Css69   GGTTGGAAATTACAATGGCCCATGAGCGTGATGGCGAACCTA-GGCACGTGTGCCATAGGTGGCACACCATCGCC
Css71   GGTTGGAAATTACAATGGCCCATGAGCGTGATGGCGAACCTA-GGCACGTGTGCCATAGGTGGCACACCATCGCC
Css74   GGTTGGAAATTACAATGGCCCATGAGCGTGATGGCGAACCTA-GGCACGTGTGCCATAGGTGGCACACCATCGCC
Css75   GGTTGGAAATTACAATGGCCCATGAGCGTGATGGCGAACCTA-GGCACGTGTGCCATAGGTGGCACACCATCGCC
                                   ↑Zn2+ -binding motif start
        531                                                                              612
           **   *****       * ***   *     * **  *     **            ******   ****  ** ** *
Css28   CCAGCTGGGTTTTATAATACCTTTTTGCTTTAGGCCTCCGCCACCATGTGACAAAAAAAAATA----GGTTCAATCAATCAAT
Css31   CCAGCTGGGTTTTATAATACCTTTTTGCTTTAGGCCTCCGCCACCATGTGACAAAAAAAAATA----GGTTCAATCAATCAAT
Css36   CCAGCTGGGTTTTATAATACCTTTTTGCTTTAGGCCTCCGCCACCATGTGACAAAAAAAAATA----GGTTCAATCAATCAAT
Css61   CCAAGTGGC----ATAATACCCTCCCGCGTTAGGACCTCGACACCACGAGACAAAAAAAAA-----GGTGAGTTTTACTAAA
Css62   CCAAGTGGC----ATAATACCCTCCCGCGTTAGGACCTCGACACCACGAGACAAAAAAAAA-----GGTGAGTTTTACTAAA
Css64   CCAAGTGGC----ATAATACCCTCCCGCGTTAGGACCTCGACACCACGAGACAAAAAAAAA-----GGTGAGTTTTACTAAA
Css65   CCAGCTGGGGTTTATAATACCTTTTTGCTTTAGGCCTCCGCCACCATGTGACAAAAAAAAAAAAAAAGGTTCAATCAATCAAT
Css66   CCAGCTGGGGTTTATAATACCTTTTTGCTTTAGGCCTCCGCCACCATGTGACAAAAAAAAAAAAAAAGGTTCAATCAATCAAT
Css67   CCAGCTGGGGTTTATAATACCTTTTTGCTTTAGGCCTCCGCCACCATGTGACAAAAAAAAAAAAAAAGGTTCAATCAATCAAT
Css68   CCAGCTGGGTTTTATAATACCTTTTTGCTTTAGGCCTCCGCCACCATGTGACAAAAAAAAATA----GGTTCAATCAATCAAT
Css69   CCAGCTGGGGTTTATAATACCTTTTTGCTTTAGGCCTCCGCCACCATGTGACAAAAAAAAAAAAAAAGGTTCAATCAATCAAT
Css71   CCAGCTGGGTTTTATAATACCTTTTTGCTTTAGGCCTCCGCCACCATGTGACAAAAAAAATAAAAAGGTTCAATCAATCAAT
Css74   CCAGCTGGGTTTTATAATACCTTTTTGCTTTAGGCCTCCGCCACCATGTGACAAAAAAAATAAAAAGGTTCAATCAATCAAT
Css75   CCAGCTGGGGTTTATAATACCTTTTTGCTTTAGGCCTCCGCCACCATGTGACAAAAAAAAAAAAAAAGGTTCAATCAATCAAT

        613                                                                           694
        ****    ***   *** ************************************************************
Css28   CAA-TTTCAACTTCTCGC-------------------------------------------------------GGGT-GGA
Css31   CAA-TTTCAACTTCTCGC-------------------------------------------------------GGGT-GGA
Css36   CAA-TTTCAACTTCTCGC-------------------------------------------------------GGGT-GGA
Css61   AGCCTTTCTGCTTCAGGCCCCCGCCACCATGTGCAGACAAAGAAGAGGGTTCAATCAATCAATCAATTCAACTGCCTGGGA
Css62   AGCCTTTCTGCTTCAGGCCCCCGCCACCATGTGCAGACAAAGAAGAGGGTTCAATCAATCAATCAATTCAACTGCCTGGGA
Css64   AGCCTTTCTGCTTCAGGCCCCCGCCACCATGTGCAGACAAAGAAGAGGGTTCAATCAATCAATCAATTCAACTGCCTGGGA
Css65   CAA-TTTCAACTTCTCGC-----------------------------------------------------------GGGA
Css66   CAA-TTTCAACTTCTCGC-----------------------------------------------------------GGGA
```

Css67 CAA-TTTCAACTTCTCGC---GGGA
Css68 CAA-TTTCAACTTCTCGC---GGGT-GGA
Css69 CAA-TTTCAACTTCTCGC---GGGA
Css71 CAA-TTTCAACTTCTCGC---GGGA
Css74 CAA-TTTCAACTTCTCGC---GGGA
Css75 CAA-TTTCAACTTCTCGC---GGGA

695 776

**** * *** *

Css28 ---GAAAGATCTCACAAAGTTGGAATTGAGTGATTGAAATTTTTAGTCCCACCCAGGTGCCCGCCACAGCCACCCAATTGGT
Css31 ---GAAAGATCTCACAAAGTTGGAATTGAGTGATTGAAATTTTTAGTCCCACCCAGGTGCCCGCCACAGCCACCCAATTGGT
Css36 ---GAAAGATCTCACAAAGTTGGAATTGAGTGATTGAAATTTTTAGTCCCACCCAGGTGCCCGCCACAGCCACCCAATTGGT
Css61 AAAAAAAGATCTCACAAAGTTGGAATTGAGTGATTGAAATTTTTAGTCCCACCCAG-CTGCCGCCACAGCCCCCCAATTGGT
Css62 AAAAAAAGATCTCACAAAGTTGGAATTGAGTGATTGAAATTTTTAGTCCCACCCAG-CTGCCGCCACAGCCCCCCAATTGGT
Css64 AAAAAAAGATCTCACAAAGTTGGAATTGAGTGATTGAAATTTTTAGTCCCACCCAG-CTGCCGCCACAGCCCCCCAATTGGT
Css65 AAAAAAAGATCTCACAAAGTTGGAATTGAGTGATTGAAATTTTTAGTCCCCCCCAGGCTGCCCCCACAGCCACCCAATTGGT
Css66 AAAAAAAGATCTCACAAAGTTGGAATTGAGTGATTGAAATTTTTAGTCCCCCCCAGGCTGCCCCCACAGCCACCCAATTGGT
Css67 AAAAAAAGATCTCACAAAGTTGGAATTGAGTGATTGAAATTTTTAGTCCCCCCCAGGCTGCCCCCACAGCCACCCAATTGGT
Css68 ---GAAAGATCTCACAAAGTTGGAATTGAGTGATTGAAATTTTTAGTCCCACCCAGGTGCCCGCCACAGCCACCCAATTGGT
Css69 AAAAAAAGATCTCACAAAGTTGGAATTGAGTGATTGAAATTTTTAGTCCCCCCCAGGCTGCCCCCACAGCCACCCAATTGGT
Css71 AAAAAAAGATCTCACAAAGTTGGAATTGAGTGATTGAAATTTTTAGTCCCCCCCAGGCTGCCCCCACAGCCACCCAATTGGT
Css74 AAAAAAAGATCTCACAAAGTTGGAATTGAGTGATTGAAATTTTTAGTCCCCCCCAGGCTGCCCCCACAGCCACCCAATTGGT
Css75 AAAAAAAGATCTCACAAAGTTGGAATTGAGTGATTGAAATTTTTAGTCCCCCCCAGGCTGCCCCCACAGCCACCCAATTGGT

777 858

* * * * ** ** **** ** *

Css28 CACACCCACCC-AATTGGCCAAACCTACCCGA-TGGGCTACGCCTACCTGGTACTCAACGATACATAAGTGGGTTTTGGTTT
Css31 CACACCCACCC-AATTGGCCAAACCTACCCGA-TGGGCTACGCCTACCTGGTACTCAACGATACATAAGTGGGTTTTGGTTT
Css36 CACACCCACCC-AATTGGCCAAACCTACCCGA-TGGGCTACGCCTACCTGGTACTCAACGATACATAAGTGGGTTTTGGTTT
Css61 CAC-CCCCCCCCAATTGGCCAACCCCCCCCGAGTGGTACGCCTCTACCTGGTACCCAACGATACATAAGTGGGTTTTGGTTT
Css62 CAC-CCCCCCCCAATTGGCCAACCCCCCCCGAGTGGTACGCCTCTACCTGGTACCCAACGATACATAAGTGGGTTTTGGTTT
Css64 CAC-CCCCCCCCAATTGGCCAACCCCCCCCGAGTGGTACGCCTCTACCTGGTACCCAACGATACATAAGTGGGTTTTGGTTT
Css65 CACACCCACCC-AATTGGCCAAACCTACCCG-GTGGGCTACACCTACCTGGTACCCAACGATACATAAGTGGGTTTTGGTTT
Css66 CACACCCACCC-AATTGGCCAAACCTACCCG-GTGGGCTACACCTACCTGGTACCCAACGATACATAAGTGGGTTTTGGTTT
Css67 CACACCCACCC-AATTGGCCAAACCTACCCG-GTGGGCTACACCTACCTGGTACCCAACGATACATAAGTGGGTTTTGGTTT
Css68 CACACCCACCC-AATTGGCCAAACCTACCCGA-TGGGCTACGCCTACCTGGTACTCAACGATACATAAGTGGGTTTTGGTTT
Css69 CACACCCACCC-AATTGGCCAAACCTACCCG-GTGGGCTACACCTACCTGGTACCCAACGATACATAAGTGGGTTTTGGTTT
Css71 CACACCCACCC-AATTGGCCAAACCTACCCG-GTGGGCTACACCTACCTGGTACCCAACGATACATAAGTGGGTTTTGGTTT
Css74 CACACCCACCC-AATTGGCCAAACCTACCCG-GTGGGCTACACCTACCTGGTACCCAACGATACATAAGTGGGTTTTGGTTT
Css75 CACACCCACCC-AATTGGCCAAACCTACCCG-GTGGGCTACACCTACCTGGTACCCAACGATACATAAGTGGGTTTTGGTTT

859 933

Css28 GCAGTTTGGGCACTCAGTCTCTAAAAGGTTCACCATCACTGGGCAATGAG**TGGGTCATAATCTGGGCATTAATCA**
Css31 GCAGTTTGGGCACTCAGTCTCTAAAAGGTTCACCATCACTGGGCAATGAG**TGGGTCATAATCTGGGCATTAATCA**
Css36 GCAGTTTGGGCACTCAGTCTCTAAAAGGTTCACCATCACTGGGCAATGAG**TGGGTCATAATCTGGGCATTAATCA**
Css61 GCAGTTTGGGCACTCAGTCTCTAAAAGGTTCACCATCACTGGGCAATGAG**TGGGTCATAATCTGGGCATTAATCA**
Css62 GCAGTTTGGGCACTCAGTCTCTAAAAGGTTCACCATCACTGGGCAATGAG**TGGGTCATAATCTGGGCATTAATCA**
Css64 GCAGTTTGGGCACTCAGTCTCTAAAAGGTTCACCATCACTGGGCAATGAG**TGGGTCATAATCTGGGCATTAATCA**
Css65 GCAGTTTGGGCACTCAGTCTCTAAAAGGTTCACCATCACTGGGCAATGAG**TGGGTCATAATCTGGGCATTAATCA**
Css66 GCAGTTTGGGCACTCAGTCTCTAAAAGGTTCACCATCACTGGGCAATGAG**TGGGTCATAATCTGGGCATTAATCA**
Css67 GCAGTTTGGGCACTCAGTCTCTAAAAGGTTCACCATCACTGGGCAATGAG**TGGGTCATAATCTGGGCATTAATCA**
Css68 GCAGTTTGGGCACTCAGTCTCTAAAAGGTTCACCATCACTGGGCAATGAG**TGGGTCATAATCTGGGCATTAATCA**
Css69 GCAGTTTGGGCACTCAGTCTCTAAAAGGTTCACCATCACTGGGCAATGAG**TGGGTCATAATCTGGGCATTAATCA**
Css71 GCAGTTTGGGCACTCAGTCTCTAAAAGGTTCACCATCACTGGGCAATGAG**TGGGTCATAATCTGGGCATTAATCA**
Css74 GCAGTTTGGGCACTCAGTCTCTAAAAGGTTCACCATCACTGGGCAATGAG**TGGGTCATAATCTGGGCATTAATCA**
Css75 GCAGTTTGGGCACTCAGTCTCTAAAAGGTTCACCATCACTGGGCAATGAG**TGGGTCATAATCTGGGCATTAATCA**

	934 ... 998
Css28	**TGAC**AGAGATTCCTGTACTTGCGGTGCTAACTAATGCATTATGGCTTCTGTTCTAAGTGATCAAT
Css31	**TGAC**AGAGATTCCTGTACTTGCGGTGCTAACTAATGCATTATGGCTTCTGTTCTAAGTGATCAAT
Css36	**TGAC**AGAGATTCCTGTACTTGCGGTGCTAACTAATGCATTATGGCTTCTGTTCTAAGTGATCAAT
Css61	**TGAC**AGAGATTCCTGTACTTGCGGTGCTAACTAATGCATTATGGCTTCTGTTCTAAGTGATCAAT
Css62	**TGAC**AGAGATTCCTGTACTTGCGGTGCTAACTAATGCATTATGGCTTCTGTTCTAAGTGATCAAT
Css64	**TGAC**AGAGATTCCTGTACTTGCGGTGCTAACTAATGCATTATGGCTTCTGTTCTAAGTGATCAAT
Css65	**TGAC**AGAGATTCCTGTACTTGCGGTGCTAACTAATGCATTATGGCTTCTGTTCTAAGTGATCAAT
Css66	**TGAC**AGAGATTCCTGTACTTGCGGTGCTAACTAATGCATTATGGCTTCTGTTCTAAGTGATCAAT
Css67	**TGAC**AGAGATTCCTGTACTTGCGGTGCTAACTAATGCATTATGGCTTCTGTTCTAAGTGATCAAT
Css68	**TGAC**AGAGATTCCTGTACTTGCGGTGCTAACTAATGCATTATGGCTTCTGTTCTAAGTGATCAAT
Css69	**TGAC**AGAGATTCCTGTACTTGCGGTGCTAACTAATGCATTATGGCTTCTGTTCTAAGTGATCAAT
Css71	**TGAC**AGAGATTCCTGTACTTGCGGTGCTAACTAATGCATTATGGCTTCTGTTCTAAGTGATCAAT
Css74	**TGAC**AGAGATTCCTGTACTTGCGGTGCTAACTAATGCATTATGGCTTCTGTTCTAAGTGATCAAT
Css75	**TGAC**AGAGATTCCTGTACTTGCGGTGCTAACTAATGCATTATGGCTTCTGTTCTAAGTGATCAAT
	↑ Zn^{2+} - binding motif end

	999 ... 1063
	* ... *
Css28	CTTCCAATTCATTCAGCAATTATAGTAAGGTGGAACATAGGAGGTATCTTATTAATCATACCCCA
Css31	CTTCCAATTCATTCAGCAATTATAGTAAGGTGGAACATAGGAGGTATCTTATTAATCATACCCCA
Css36	CTTCCAATTCATTCAGCAATTATAGTAAGGTGGAACATAGGAGGTATCTTATTAATCATACCCCA
Css61	CTTCCAATTCATTCAGCAATTGTAGTAAGGTGGAACATAGGAGGTATCTTATTAATCATCCCCCA
Css62	CTTCCAATTCATTCAGCAATTGTAGTAAGGTGGAACATAGGAGGTATCTTATTAATCATCCCCCA
Css64	CTTCCAATTCATTCAGCAATTGTAGTAAGGTGGAACATAGGAGGTATCTTATTAATCATCCCCCA
Css65	CTTCCAATTCATTCAGCAATTATAGTAAGGTGGAACATAGGAGGTATCTTATTAATCATACCCCA
Css66	CTTCCAATTCATTCAGCAATTATAGTAAGGTGGAACATAGGAGGTATCTTATTAATCATACCCCA
Css67	CTTCCAATTCATTCAGCAATTATAGTAAGGTGGAACATAGGAGGTATCTTATTAATCATACCCCA
Css68	CTTCCAATTCATTCAGCAATTATAGTAAGGTGGAACATAGGAGGTATCTTATTAATCATACCCCA
Css69	CTTCCAATTCATTCAGCAATTATAGTAAGGTGGAACATAGGAGGTATCTTATTAATCATACCCCA
Css71	CTTCCAATTCATTCAGCAATTATAGTAAGGTGGAACATAGGAGGTATCTTATTAATCATACCCCA
Css74	CTTCCAATTCATTCAGCAATTATAGTAAGGTGGAACATAGGAGGTATCTTATTAATCATACCCCA
Css75	CTTCCAATTCATTCAGCAATTATAGTAAGGTGGAACATAGGAGGTATCTTATTAATCATACCCCA

	1064 ... 1128
	* *
Css28	CAATGCATTCTCAATGAACCCCTGTGCACAGATATTGTTTCCCCTGCAGTTTGTGGAAATGAACT
Css31	CAATGCATTCTCAATGAACCCCTGTGCACAGATATTGTTTCCCCTGCAGTTTGTGGAAATGAACT
Css36	CAATGCATTCTCAATGAACCCCTGTGCACAGATATTGTTTCCCCTGCAGTTTGTGGAAATGAACT
Css61	CAATGCATTCTCAATGACCCCTTGTGCACAGATATTGTTTCCCCTGCAGTTTGTGGAAATGAACT
Css62	CAATGCATTCTCAATGACCCCTTGTGCACAGATATTGTTTCCCCTGCAGTTTGTGGAAATGAACT
Css64	CAATGCATTCTCAATGACCCCTTGTGCACAGATATTGTTTCCCCTGCAGTTTGTGGAAATGAACT
Css65	CAATGCATTCTCAATGAACCCCTGTGCACAGATATTGTTTCCCCTGCAGTTTGTGGAAATGAACT
Css66	CAATGCATTCTCAATGAACCCCTGTGCACAGATATTGTTTCCCCTGCAGTTTGTGGAAATGAACT
Css67	CAATGCATTCTCAATGAACCCCTGTGCACAGATATTGTTTCCCCTGCAGTTTGTGGAAATGAACT
Css68	CAATGCATTCTCAATGAACCCCTGTGCACAGATATTGTTTCCCCTGCAGTTTGTGGAAATGAACT
Css69	CAATGCATTCTCAATGAACCCCTGTGCACAGATATTGTTTCCCCTGCAGTTTGTGGAAATGAACT
Css71	CAATGCATTCTCAATGAACCCCTGTGCACAGATATTGTTTCCCCTGCAGTTTGTGGAAATGAACT
Css74	CAATGCATTCTCAATGAACCCCTGTGCACAGATATTGTTTCCCCTGCAGTTTGTGGAAATGAACT
Css75	CAATGCATTCTCAATGAACCCCTGTGCACAGATATTGTTTCCCCTGCAGTTTGTGGAAATGAACT
	↑Spacer/Disintegrin start (Hite et al., 1994)

	1129 ... 1147
Css28	TTTGGAGGCGGGAGAAGAA
Css31	TTTGGAGGCGGGAGAAGAA
Css36	TTTGGAGGCGGGAGAAGAA
Css61	TTTGGAGGCGGGAGAAGAA

Css62	TTTGGAGGCGGGAGAAGAA
Css64	TTTGGAGGCGGGAGAAGAA
Css65	TTTGGAGGCGGGAGAAGAA
Css66	TTTGGAGGCGGGAGAAGAA
Css67	TTTGGAGGCGGGAGAAGAA
Css68	TTTGGAGGCGGGAGAAGAA
Css69	TTTGGAGGCGGGAGAAGAA
Css71	TTTGGAGGCGGGAGAAGAA
Css74	TTTGGAGGCGGGAGAAGAA
Css75	TTTGGAGGCGGGAGAAGAA
